# Origin and distribution of the BRCA2-8765delAG mutation in breast cancer

**DOI:** 10.1186/1471-2407-7-132

**Published:** 2007-07-19

**Authors:** Grazia Palomba, Antonio Cossu, Eitan Friedman, Mario Budroni, Antonio Farris, Antonio Contu, Marina Pisano, Paola Baldinu, Maria C Sini, Francesco Tanda, Giuseppe Palmieri

**Affiliations:** 1 Istituto di Chimica Biomolecolare-Consiglio Nazionale Ricerche, Sassari, Italy; 2 Servizio di Anatomia Patologica, Azienda USL1, Sassari, Italy; 3 Chaim Sheba Medical Center, Tel-Hashomer, Israel; 4 Centro Epidemiologico Multizonale, Azienda USL1, Sassari, Italy; 5 Università degli Studi di Sassari, Italy; 6 Ospedale Civile, Azienda USL1, Sassari, Italy

## Abstract

**Background:**

The *BRCA2*-8765delAG mutation was firstly described in breast cancer families from French-Canadian and Jewish-Yemenite populations; it was then reported as a founder mutation in Sardinian families. We evaluated both the prevalence of the *BRCA2*-8765delAG variant in Sardinia and the putative existence of a common ancestral origin through a haplotype analysis of breast cancer family members carrying such a mutation.

**Methods:**

Eight polymorphic microsatellite markers (D13S1250, centromeric, to D13S267, telomeric) spanning the *BRCA2 *gene locus were used for the haplotype analysis. Screening for the 8765delAG mutation was performed by PCR-based amplification of *BRCA2*-exon 20, followed by automated sequencing.

**Results:**

Among families with high recurrence of breast cancer (≥ 3 cases in first-degree relatives), those from North Sardinia shared the same haplotype whereas the families from French Canadian and Jewish-Yemenite populations presented distinct genetic assets at the BRCA2 locus. Screening for the *BRCA2*-8765delAG variant among unselected and consecutively-collected breast cancer patients originating from the entire Sardinia revealed that such a mutation is present in the northern part of the island only [9/648 (1.4%) among cases from North Sardinia versus 0/493 among cases from South Sardinia].

**Conclusion:**

The *BRCA2*-8765delAG has an independent origin in geographically and ethnically distinct populations, acting as a founder mutation in North but not in South Sardinia. Since *BRCA2*-8765delAG occurs within a triplet repeat sequence of AGAGAG, our study further confirmed the existence of a mutational hot-spot at this genomic position (additional genetic factors within each single population might be involved in generating such a mutation).

## Background

Breast Cancer (BC) is the most common malignancy among women in industrialized countries. Germline mutations in BRCA1 (MIM 113705) and BRCA2 (MIM 600185) genes account for cancer predisposition in majority of families with BC recurrence. Positive family history is indeed recognized as a high risk factor for developing the disease; about 10% of all breast cancers arise in individuals carrying a germline mutation in one of such two genes [1].

According to the Breast Cancer Information Core (BIC) database [2], the majority of germline alterations identified in *BRCA1 *and *BRCA2 *is unique (57% and 63%, respectively); the remaining ones are recurrent founder mutations which have been described in different ethnic groups and populations [3]. Mutations that are observed repeatedly associated with a common haplotype are likely to have descended from a common ancestor, and are referred to as "founder mutations". Although at different rates, *BRCA1 *and *BRCA2 *founder mutation have been detected in genetically homogeneous populations, such as the Icelanders [4,5], the Ashkenazi Jews [6], the Finns [7], and the French-Canadians [8].

The *BRCA2*-8765delAG mutation was described as a founder mutation in Jewish-Yemenite families [9], as well as in French-Canadian families [8,10]. Our group has also described the *BRCA2*-8765delAG variant as a founder mutation in about 12% breast cancer families originating from different villages of North Sardinia [11-13]. Although the age of the BRCA2-8765delAG variant has not yet been determined, this mutation appears on an ancient haplotype [11]. Moreover, the BRCA2-8765delAG mutation has been demonstrated to be present in all affected individuals from the four (57%) out of seven breast cancer families who shared an identical-by-descent haplotype within North Sardinia [11]. A previous study has also reported the comparison of haplotypes of French Canadians and Yemenite Jewish mutation carriers, indicating the independent origins of carriers of the BRCA2-8765delAG mutation in these two populations [14].

Overall, this mutation has been reported forty-two times into the Breast Cancer Information Core database, and it has been found to occur in populations throughout Europe and North America. No information is however available about the question whether *BRCA2*-8765delAG may be an ancient or common mutation in Mediterranean area (which Sardinia and Israel belong to). In this sense, we performed a haplotype analysis among breast cancer families carrying the BRCA2-8765delAG mutation from Sardinian and Jewish-Yemenite populations (also referring to data among French-Canadian families) as well as definitely determined the prevalence of such a variant among unselected breast cancer cases originating from the entire Sardinia island.

## Methods

### Patients and families

Families containing probands with histologically-proven diagnosis of breast cancer and carrying the *BRCA2*-8765delAG mutation were recruited from clinics at the University of Sassari and Azienda Unità Sanitaria Locale 1 of Sassari (which represent the principal medical institutions accounting for cancer patients from the central and northern parts of the island) and from familial cancer genetics clinics in Israel; DNA samples of two French-Canadian families carrying the BRCA2-8765delAG were previously provided by P. Tonin. Sardinian families were unrelated since they did not present any common ancestor after evaluation of pedigrees up to 1700.

All information regarding the recurrence of cancers in family, age at cancer diagnosis, and age at death or current age were collected after obtaining a written informed consent. Most of the information were verified by analysis of the Hospital records; all cancer diagnoses were confirmed by pathology reports. After the patients were informed about the aims and limits of the study, blood samples were obtained with their written consent. DNA samples were collected from at least one affected member of each family.

Appropriate institutional informed consent guidelines were followed for all recruited patients. The study was reviewed and approved by the ethical review boards of both participating institutions (University and Azienda USL1 of Sassari).

### DNA analysis

Haplotype analysis was performed using nine polymorphic microsatellite repeat markers, three intragenic (D13S260, D13S1701, and D13S171) and the remaining flanking the *BRCA2 *gene (D13S1250, D13S1299, D13S1246, D13S289, D13S310 and D13S267). For these markers, heterozygosity varied from 69% to 82%; primer sequences and PCR conditions for amplification were as indicated into the Human Genome Database [15]. Marker loci were ordered as in Ensembl map [16] (Table 1). Alleles were numbered according to size for each microsatellite marker.

**Table 1:** Results from haplotype analysis

**Cromo some**	**Ensembl Map (Mb)**	**Marker**	**North Sardinia**	**Jewish-Yemenite**		**French-Canadian**	
				
				Fam.1	Fam.2	Fam.1	Fam.2
13q12.3	27.80	D13S1250	8	8	5	n.t.	n.t.
	29.45	D13S1299	3	3	2	n.t.	n.t.
	30.00	D13S1246	5	4–8	1	n.t.	n.t.
	30.15	D13S289	3	3	3	n.t.	n.t.
13q13.1	31.35	D13S260	8	8	3	7	7
	**31.80**	**BRCA2**					
	32.05	D13S1701	6	5	5	8	8
	32.15	D13S171	10	3–4	4	4–11	4–6
	32.65	D13S310	5	5	3	n.t.	n.t.
13q13.2	33.15	D13S267	2	5–8	8	n.t.	n.t.

The genomic position of the *BRCA2 *gene is indicated. n.t., not tested

Briefly, DNA samples from 32 individuals in 7 families (three from North-Sardinia, two from Israel and two from Canada) carrying the *BRCA2*-8765delAG mutation were genotyped. All primers labelled with fluorescein derivatives (5'-6-FAM, or 5'-HEX) were from MWG-Biotech (Ebersberg, Germany). For each sample, 50 ng of genomic DNA was amplified using 200 μmol/L deoxynucleotide triphosphates, 0.4 μmol/L primers, 1.5 mmol/L MgCl_2_, and 0.625 units of Taq DNA Polymerase, according to standard PCR procedures. Microsatellite genotyping was carried out on an automated ABIPRISM 3100 DNA sequencer (Applied Biosystems, Foster City, CA) and PCR fragments were analysed using the Genescan 3.7.1^® ^software.

Genomic DNA for mutational screening of 1,141 unselected breast cancer patients was isolated from both peripheral blood samples (N = 691) and archival paraffin-embedded tissues (N = 450), using standardized procedures. To avoid any bias, paraffin-embedded normal tissues from breast cancer patients were consecutively collected; all cases were thus included regardless of age at diagnosis, family history status, and disease features. Sardinian origin was ascertained in all cases through genealogical studies. For mutation screening, exon 20 of the *BRCA2 *gene was amplified and PCR products were directly sequenced using an automated fluorescence-cycle sequencer.

## Results

**Figure 1:**
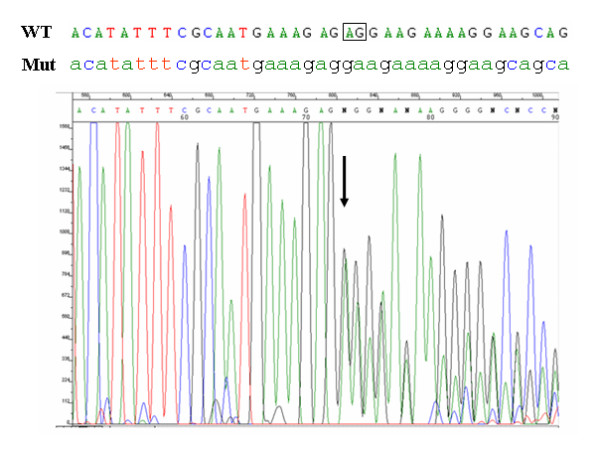
***BRCA2*-8765delAG mutation**. Electropherogram shows the nucleotide sequence at the genomic level in a positive breast cancer patient. The wild-type (WT) and the mutant (Mut) nucleotide sequences have been reported (the deleted AG has been boxed). Arrow indicates the mutation position.

**Figure 2:**
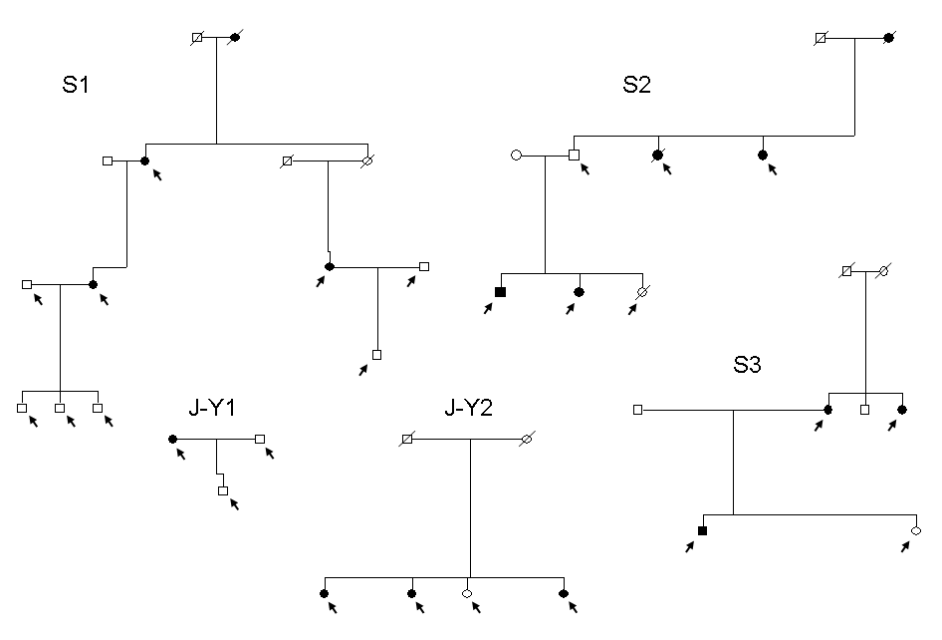
**Pedigrees of analyzed *BRCA2*-8765delAG positive families from Sardinian and Jewish-Yemenite populations**. Arrows indicate the family members who underwent haplotype analysis. S and J-Y, Sardinian and Jewish-Yemenite families (respectively).

For haplotype analysis, breast cancer families carrying the *BRCA2*-8765delAG mutation and originating from North-Sardinia (N = 3), Israel (N = 2), and French area of Canada (N = 2) were included into the study. Among the seven families, 32 members, including 16 probands with histologically-proven diagnosis of breast cancer, were genotyped with markers spanning the *BRCA2 *locus at chromosome 13q12-q13. All families had at least three individuals diagnosed with breast cancer in first-degree relatives and presence of the *BRCA2*-8765delAG mutation in all family probands from the three populations was confirmed by direct sequencing (Figure 1). The *BRCA2*-8765delAG mutation was absent in blood DNA from 103 unrelated healthy individuals (corresponding to 206 chromosomes), originating from the same geographical area and used as normal controls. Pedigrees of analyzed BC families from North Sardinia and Israel are shown in Figure 2. For both French-Canadian families, one proband along with the two parents from each family was analyzed.

In Sardinian families, an identical haplotype was inferred by genotyping all affected family members carrying the *BRCA2*-8765delAG mutation with marker loci D13S1250 to D13S267 (centromeric to telomeric) (Table 1). This unique haplotype (8-3-5-3-8-10-5-2) among BC families from North Sardinia was not found in 80 control chromosomes from the same geographical area. Surprisingly, the two Jewish-Yemenite families presented two different haplotypes; one of them only, Fam.1, shared majority of the alleles (5/9; 56%) observed into the Sardinian series (Table 1). For the remaining French-Canadian families, haplotype analysis was carried out for the three closely flanking BRCA2 marker loci only (D13S260, D13S1701, and D13S171), due to the low amount of the available germline DNA. Although with such a limited number of markers, the inferred haplotype of the French-Canadian cases was consistent in families from the same population but different when compared with familial cases from both the Sardinian and Jewish-Yemenite populations (Table 1). Overall, the *BRCA2*-8765delAG mutation was found to occur associated with distinct haplotypes into the three analyzed populations.

**Figure 3:**
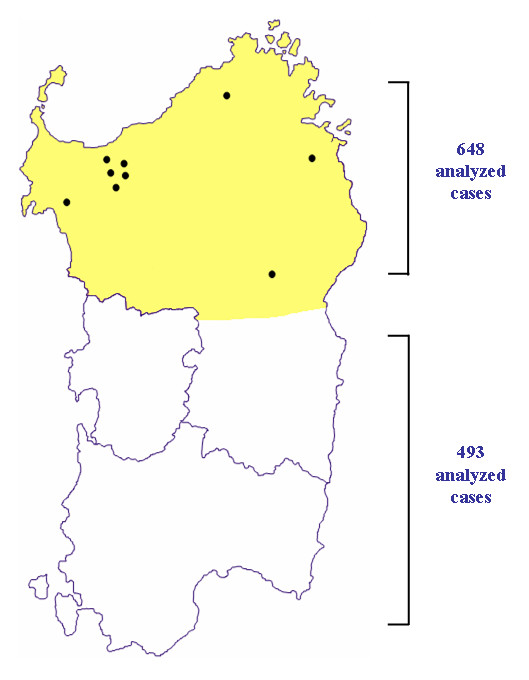
**Distribution of breast cancer patients carrying the *BRCA2*-8765delAG mutation**. Geographical areas of the island from which patients were collected are indicated (the two colours correspond to the northern and southern part of the island). Dots indicate the origin of patients presenting the *BRCA2*-8765delAG mutation.

To evaluate the prevalence of BRCA2-8765delAG in breast cancer cases across the entire Sardinia, we screened genomic DNA obtained from both peripheral blood and paraffin-embedded normal tissues (see Methods) of 1,141 unselected and consecutively-collected BC patients originating from either the northern (N = 648) or southern (N = 493) part of the island. The 2-bp deletion in exon 20 of the *BRCA2 *gene was detected by direct automated sequencing of the correspondent PCR products (Figure 1). The *BRCA2*-8765delAG mutation was found in 9/648 (1.4%) cases from North Sardinia and 0/493 cases from South Sardinia (Figure 3). Interestingly, all the nine breast cancer patients found positive to *BRCA2*-8765delAG were from families with at least three affected members in first-degree relatives [whereas familial recurrence of breast cancer was observed in 87 (13.5%) out of 644 mutation-negative patients, whose information on family history for cancer was available], further confirming that occurrence of the BRCA2 mutations is strictly associated with the presence of a higher familial recurrence of the disease.

## Discussion

The unique haplotype common to all three breast cancer families from North Sardinia indicated the *BRCA2*-8765delAG as a founder mutation in this geographical area. Haplotype analysis revealed that Sardinian mutation carriers shared majority of allele markers with only one of the two Jewish-Yemenite families; the remaining Jewish-Yemenite family and the French-Canadian families did not present the same genotypic asset (see Table 1).

In the BIC database [2], the *BRCA2*-8765delAG variant is a recurrent mutation occurring in a AG-rich sequence of the *BRCA2 *gene. Recombination events or marker mutations could theoretically account for haplotype divergence from a single, original haplotype but seem not to be likely explanations here. Indeed, our results indicate that carriers of the BRCA2-8765delAG variant may have an independent origin and, although already suggested for French-Canadians and Yemenite-Jews [14], allow to exclude the presence of a common ancestor in Mediterranean area also. Therefore, one could hypothesize the existence of a mutational hot-spot at this genomic position within the *BRCA2 *gene locus. Since the mutation is an AG-deletion in a stretch of AGAGAG, the chance of recurrent mutations at this site might be higher than that in a site having a single AG. On this regard, differences into the genetic background may be due to a replication slippage resulting in the loss of repeat units during meiosis or recombination events (putatively dependent on the simultaneous presence of additional genetic factors or, more in general, favouring genomic backgrounds). As a confirmation of this hypothesis, other authors have described different nucleotide deletions at this genomic level among families with breast and/or ovarian cancer [10,14,17,18]. Nevertheless, one should also take into account that a deletion of AG in a triplet repeat sequence of AGAGAG does not allow to accurately determine whether the mutations that occur in the different populations are really identical.

Considering the incidence of the *BRCA2*-8765delAG variant among the unselected patients from the Sardinian population, our extensive screening clearly indicated that such a mutation is recurrent in North Sardinia (confirming its role as founder mutation in this part of the island [11-13]) but absent in South Sardinia. The Sardinia island has a relatively small, isolated, and genetically homogeneous population with a high rate of inbreeding; several founder effects have been already demonstrated for monogenic or complex diseases in this population [19]. For breast cancer, heterogeneous genetic backgrounds seem to instead account for such deep differences into the *BRCA2 *gene involvement. As we recently demonstrated for malignant melanoma [20], these findings further support the hypothesis that patients' origin (even in so close geographical areas) may determine different susceptibility roles of the candidate cancer genes and, for the same gene, different mutation rates might be observed.

As we previously described [11-13], the prevalence of the *BRCA2*-8765delAG mutation in breast cancer families was lower than that of other *BRCA1-2 *founder mutations observed in different genetically-homogeneous populations (three BRCA1 and a single *BRCA2 *founder mutations account for vast majority of breast cancer families among Ashkenazi Jews [21] and Icelanders [22], respectively). Considering the screening of consecutively-collected unselected BC cases, the fraction of 1.4% *BRCA2 *mutation carriers is analogously low in breast cancer patients from North Sardinia (again, a quite sharp contrast between our frequencies and those observed in unselected cases from either Ashkenazi Jewish [23,24] and Icelandic [22,25] populations was observed).

In conclusion, our study allowed to assess that *BRCA2*-8765delAG, arising in conjunction with several and clearly distinct genetic haplotypes, may be generated in an independent manner and at a frequency higher than other *BRCA2 *mutations among breast cancer families world-wide. A clinically relevant implication is that such a recurrent mutation should be included in targeted *BRCA2 *mutation screening panels in any population, irrespective of ethnic origin. Finally, our heterogeneous data from the screening of breast cancer patients belonging to a so-recognized genetically-homogeneous population, can be further considered as indicative that both role and mutation prevalence of any candidate cancer gene needs to be evaluated in each geographical area.

## Competing interests

The author(s) declare that they have no competing interests.

## Authors' contributions

GRP participated in the design of the study and performed the molecular analyses. ACOS performed the data management. EF participated in the families' collection. MB participated in the analysis and interpretation of data. AF participated in the patients' collection. ACON participated in the patients' collection. MP performed some haplotype analyses. PB performed some screening analyses. MCS performed some screening analyses. FT participated in the design of the study. GIP conceived of the study and drafted the manuscript.

## Pre-publication history

The pre-publication history for this paper can be accessed here:


